# In vivo imaging of immediate early gene expression dynamics segregates neuronal ensemble of memories of dual events

**DOI:** 10.1186/s13041-021-00798-3

**Published:** 2021-06-29

**Authors:** P. Meenakshi, S. Kumar, J. Balaji

**Affiliations:** Centre for Neurosciences, Indian Institute of Science, Bangalore, 560012 India

**Keywords:** cfos expression, Neuronal ensembles, Dual context exposure, Retrosplenial cortex, IEGs, Two-photon, In vivo imaging

## Abstract

Identification of neurons undergoing plasticity in response to external stimuli is one of the pertinent problems in neuroscience. Immediate early genes (IEGs) are widely used as a marker for neuronal plasticity. Here, we model the dynamics of IEG expression as a consecutive, irreversible first-order reaction with a limiting substrate. First, we develop an analytical framework to show that such a model, together with two-photon in vivo imaging of IEG expression, can be used to identify distinct neuronal subsets representing multiple memories. Using the above combination, we show that the expression kinetics, rather than intensity threshold, can be used to identify neuronal ensembles responding to the presentation of two events in vivo. The analytical expression allowed us to segregate the neurons based on their temporal response to one specific behavioural event, thereby improving the ability to detect plasticity related neurons. We image the retrosplenial cortex (RSc) of cfos-GFP transgenic mice to follow the dynamics of cellular changes resulting from contextual fear conditioning behaviour, enabling us to establish a representation of context in RSc at the cellular scale following memory acquisition. Thus, we obtain a general method that distinguishes neurons that took part in multiple temporally separated events by measuring fluorescence of individual neurons in live mice.

**Supplementary Information:**

The online version contains supplementary material available at 10.1186/s13041-021-00798-3.

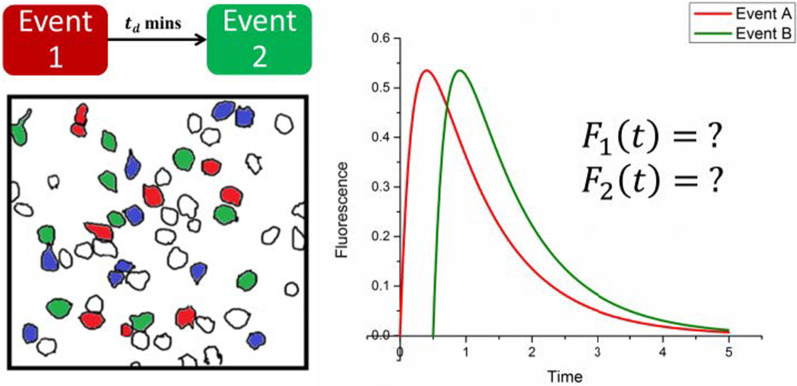

## Introduction

Identifying the neuronal ensemble that possibly encodes memory and understanding the changes occurring in the memory circuits over time are pertinent problems in neuroscience research [8, 19, 20, 31]. Immediate early genes are rapidly and transiently expressed in response to neuronal activity and hence used as a marker for plasticity. Generally, to identify the temporal coupling of IEG expression to different behaviours or events, it requires as many distinct molecular labels as the number of events that are being followed.

Cellular compartment analysis of temporal activity by fluorescent in situ hybridization (catFISH) [16–18] initially exploited the unique transport kinetics of Arc mRNA from the nucleus to the cytoplasm, and later has been improved to use the intronic regions of Arc that have faster kinetics along with other IEGs such as Homer [39] to dissect the temporal engagement of neurons. Despite such improvements the method is limited to in vitro identification. Another technique uses a combination of IEG promoter-based expression and a modified Tet-OFF system to achieve the labelling of two distinct populations of neurons [29]. In both cases, the visualisation of the signal is done post hoc in vitro, limiting the investigation of the neuronal population to a snapshot at any given time. Such methods cannot follow an ensemble of neurons and observe their evolution longitudinally.

Neuronal activity indicators such as genetically encoded calcium or voltage indicators (GECIs or GEVIs) are reporters of neuronal firing in sub-millisecond time scales [21, 23]. However, they do not necessarily report the plastic events that occur in response to these firings. Further, they require high-speed imaging with temporal resolution matching the indicators' response time, requiring the imaging be done on awake behaving mice. The slow kinetics of IEG expression enables following cellular plastic events in anesthetised mice after behavioural training [2, 9, 10, 37].

Here, we propose to follow the kinetics of IEG expression in vivo to identify distinct neuronal subsets, each corresponding to distinct events. First, we analytically describe the IEG protein expression dynamics as a function of time. In case of a fluorophore expressed under an IEG promoter, the kinetics can be followed by measuring the fluorescence as a function of time. Among the various IEGs, cfos expression is a widely used marker for cellular activity [13]. We validate our model using data obtained from cfos-eGFP [6] and cfos-shGFP [29] transgenic mice under two conditions: bicuculline-induced seizure and behavioural induction of IEG following contextual exposure. Following this, the kinetics of IEG-fluorescence expression is used to determine when the neuron was activated, enabling us to distinguish between neuronal populations that took part in different events that are separated in time. Thus, we hypothesize that the memory engram of multiple events can be identified using the expression dynamics of an immediate early gene and experimentally show that we can do so for two events.

Recent and emerging evidences suggest RSc plays a vital role in encoding context-related information [3–5, 24, 25]. Clustered addition of spines is observed in RSc when contextual training is carried out across multiple sessions [14]. Similarly, the inactivation of RSc prevents contextual retrieval in mice and water maze post-training [12, 28]. Preferential activation of RSc during spatial navigation has also been reported [11]. Interestingly, it is also shown that in schema-dependent encoding of related events, RSc is engaged only during the encoding of new learning related to prior information but not during encoding of completely novel information [35]. More importantly, RSc lesion in rats abolishes their ability to resolve context-based conflicts [27]. Thus, all these studies suggest RSc plays an active role during the context-based behaviour, although the nature of this role is unclear. Given its function, we reasoned that RSc might maintain contextual information in the form of independent representations and its interrelations. If such contextual interrelations have cellular representations in RSc, we would be able to locate them using our method. This is possible as the method described here, can identify, and longitudinally follow the activated cellular ensembles in vivo. Thus, in this study we simultaneously probe representation for a context, how it changes across time, and when a new context is introduced close in time.

## Results

### Consecutive first-order kinetics describes IEG protein expression following a behavioural event

**Fig. 1:**
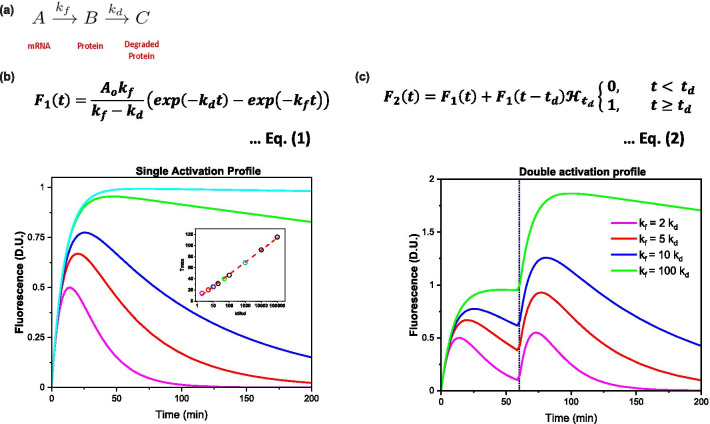
Analytical description of an IEG expression in response to plasticity related events. **a** A simple consecutive reaction kinetics for the mRNA (A), protein (B) and degraded protein (C) describes the response to a plasticity signal. The reaction is assumed to proceed with first order forward reaction rate constants *k*_*f*_ and *k*_*d*_ for the synthesis and degradation of proteins, respectively. **b** Solving the coupled differential equations of the sequential chemical reaction described in (**a**), we get an expression (Eq. 1) for F_1_(t) that describes the fluorescence intensity corresponding to IEG coupled fluorophore at any given time ‘t’ as a difference of two exponential terms with rate constants, *k*_*f*_ and *k*_*d*_. The lines are the simulated response functions for five values of k_f_/k_d_ ratios with parameter A and k_f_ set to 1D.U and 0.1 min^−1^. Time to maximal response, one of the key parameters necessary to time the neuronal tagging is plotted for these set of ratios as a scatter plot in the inset. The colour of the open circles corresponds to their respective solid lines. The red dashed line is a straight line fit of these scatter plot. **c** Similarly, we describe Eq. 2 for a neuron that got activated twice where A, k_f_, k_d_ are as previously described and t_d_ is the time of second activation event. Equation 2 is simulated (solid lines) to show the response for four ratios of *k*_*f*_*/k*_*d*_ with parameter A set to 1D.U., and the time gap between the two events (t_d_) is set to 60 min as indicated by the black dotted line

**Fig. 2:**
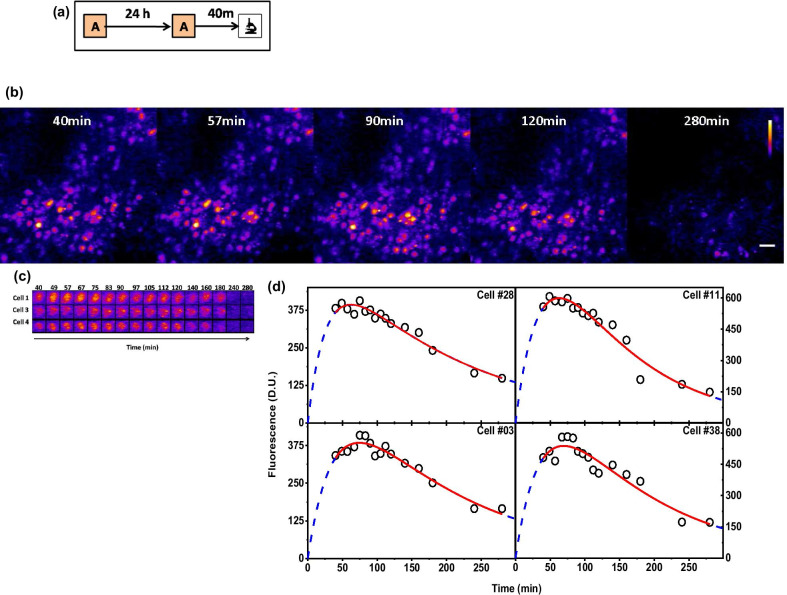
Quantification of behaviourally induced cfos-eGFP protein expression fits well to derived equation. **a** Behavioural schematic used for inducing cfos expression. Transgenic mice are trained in context A then made to recall the training context (Context A) after 24 h. The resulting activation of IEGs is followed, through in-vivo imaging of the RSc in anesthetised mice. **b** Maximum intensity projection of a stack of images corresponding to 200 × 200 × 200 μm region obtained at different time points. The cfos-eGFP signal is localised to the nucleus and hence the activated neurons appear as quasi circular regions of bright pixels with a diameter of ~ 20-pixel units. Snapshots RSc area shown are that of time points 40, 57, 90,120 and 280 min. The scale bar in the image is 20 microns. **c** Three representative image ROIs centred around cell “#01”, “#03,” and “#04” in cfos-EGFP transgenic mice across different time points are shown as image matrix. **d** The quantitative measure of fluorescence and hence the cellular expression profile of four representative cells in **b** along with their fits to Eq. 1 are shown here. The open circles represent the amplitude of the cellular activity from a neuron at a given time. The red line is the fit of this data to Eq. 1. A good agreement of the fit to the observed data (Adj. R Sq > 0.92) indicates that our model is consistent with the observed cellular response. Blue dotted line extends the fits and spans the entire x-axis. See Table 1 for fit parameters

**Fig. 3:**
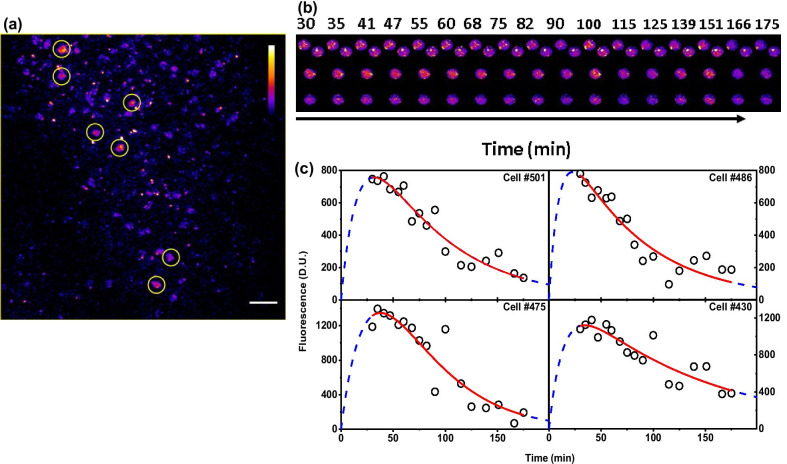
Quantification of behaviourally induced cfos-shGFP fluorescence describing the protein expression profile fits well to derived equation (Eq. 1). indicating that the analytical equation generalises to different fluorophore constructs. **a** Images from the RSc region of the cfos-shGFP transgenic mice reveal the circular bright nuclei at 90 min. The image is one of the 200 × 200 microns optical section. The scale bar represents 20 microns. **b** Four representative image ROIs centred around cells “#501”, “#486”, “#475” and “#430” in cfos-shGFP transgenic mice across different time points respectively. **c** Are the corresponding quantitative measure of cellular expression profile of cells in **b** along with their fits to Eq. 1. The open circles represent the amplitude of the cellular activity from a neuron at a given time. The red line is the fit of this data to Eq. 1. A good agreement of the fit to the observed data (Adj. R Sq > 0.79) indicates that our model is consistent with the observed cellular response. Blue dotted line extends the fits and spans the entire x-axis as explained in Fig. 2. See Table 2 for fit parameters

We investigate whether the derived analytical expression (Fig. 1, see “Appendix: Theory”) describes IEG protein expression in vivo in cfos-eGFP transgenic mice. In these mice, eGFP is expressed under the cfos promoter where the level of fluorescence indicates the cfos protein concentration in the nucleus of the neuron. We use these mice to study the expression kinetics of cfos in response to seizures (via bicuculline administration [26]) and context exposure. Immediately after the seizure or context exposure, mice are subjected to in vivo imaging of the RSc as, a function of time (Additional file 3: Fig. S3a, Fig. 2a). Our imaging time points range from 20 to 280 min, typically these images are ~ 10 min apart during the initial phase and later adjusted to capture the slower decline in fluorescence with minimal number of image acquisitions. This results in a four-dimensional image stack consisting of three spatial and one temporal dimension. We note that long periods of anaesthesia can alter the IEG expression. However, for this study we limit the anaesthesia administration to two imaging sessions in all the mice (except 1) and duration of each administration to 180 min (except the single administration that lasted for 280 min). Further we also note that IEG expression profile induced by constant presence of anaesthesia will have different time profile compared to a profile triggered by distinct behavioural event. All throughout this series, we ensure that the imaging setup parameters, namely the incident power (30 mW), pulse width (~ 100 fs), excitation wavelength (900 nm), and gain of the detection system are kept constant. The resulting images show cfos-eGFP fluorescence as circular concentrated regions of higher intensities spread across the field of view. Figures 2b and 3a shows a representative snapshot of cfos-eGFP and cfos-shGFP fluorescence image of RSc respectively.

The fluorescence from these individual nuclei identified as circular ROIs can be seen to increase initially, reaches a peak, and then decays to baseline values. This temporal profile is consistent across both the seizure and behaviourally induced cfos expression (Additional file 3: Fig. S3b, Fig. 2c). The fluorescence response from few representative cells as a time series is presented in Figs. 2c and 3b. The fluorescence values from these cells are extracted as explained in Methods (Additional file 1: Fig. S1). For each imaging time point, the identified ROIs are loaded and re-centred based on the centre of mass to correct any misalignment among images of different time points. Thus, the coordinates of the measurement ROIs are shifted rather than aligning the entire image (See “Materials and methods”). This enables us to measure the fluorescence intensity using the directly observed raw intensity values without having to align the images using image registration methods. These fluorescence values are then used to obtain the cellular responses/activity as a time series. These values are then fit to the Eq. 1.

We employ the above procedure in two different sets of mice: cfos-eGFP and cfos-shGFP. In both these cases, we extract and use the fluorescence to obtain the cellular activity profiles and fit to Eq. 1. Figure 2d shows the cellular activity profile (open circles) and their fits (solid line) of four representative neurons of cfos-eGFP transgenic mice in response to context exposure. Similarly, Fig. 3c shows the fit of fluorescence obtained from neurons of cfos-shGFP mice. Additional file 3: Fig. S3c shows the cellular activity profile of four representative cells and their fit to Eq. 1 in response to seizures. The fit parameters are provided in Tables 1, 2, Additional file 3: Table S3, respectively. We see a good agreement of our model with the observed data. Since adjusted R-square (Adj. RSq) estimates the quality of a fit, we use this in our analysis to estimate the fraction of cells that fit with an Adj. R Sq. > 0.5. We see that of the 2527 number of ROIs identified as cells from five mice, ~ 75% of the cells shows a fit with an Adj. R Sq. greater than 0.5. A good agreement of the experimental data with our model in both these mice lines following seizure as well as the behavioural activation suggests that our method can identify the neuronal ensemble that represents activation, thus enabling us to use this as a criterion for identifying a cell that got activated. Further, the fluorescence signal as a function of time is well described by the analytical expression irrespective of the transgenic mice used. Thus, it supports the hypothesis that the analytical equation can be generalised to the protein expression kinetics of other IEGs (e.g., arc, zif).

**Table 1:** Summary of fit parameters of cfos-eGFP expression in response to single context exposure data fit to Eq. 1

Fig No	Cell No	Amplitude (D.U.)	Error in Amplitude	k_f_ (min^−1^)	Error in k_f_	K_d_ (min^−1^)	Error in k_d_	Adj-R-sq	AIC
Figure 2(d)	Cell #28	541	26.39433	0.03675	0.00495	0.00514	5.31E−04	0.95524	97.85696
Figure 2(d)	Cell #11	1050	148.97179	0.02585	0.00614	0.00895	0.00182	0.94341	123.23644
Figure 2(d)	Cell #03	596	50.12666	0.02569	0.00406	0.00592	8.67E−04	0.93926	101.22503
Figure 2(d)	Cell #38	461	27.40859	0.03473	0.0054	0.00487	6.20E−04	0.93042	98.06544

**Table 2:** Summary of fit parameters of cfos-shGFP expression in response to single context exposure data fit to Eq. 1

Fig No	Cell No	Amplitude (D.U.)	Error in Amplitude	k_f_ (min^−1^)	Error in k_f_	K_d_ (min^−1^)	Error in k_d_	Adj-R-sq	AIC
Figure 3c	Cell #501	1183	148.68142	0.0593	0.02149	0.01404	0.00272	0.91592	150.73661
Figure 3c	Cell #486	1089	104.53632	0.10002	0.07986	0.014	0.00265	0.86964	157.76146
Figure 3c	Cell #475*	3663	416.218	0.02647	-	0.02647	0.0036	0.87058	184.62851
Figure 3c	Cell #430	1471	157.13412	0.07198	0.03514	0.0079	0.00176	0.79118	172.29792

^*^The k_f_ and k_d_ values for these cells were shared

### Distribution of fit parameters demonstrate that the rise kinetics of cfos-eGFP and cfos-shGFP expression are similar

**Fig. 4:**
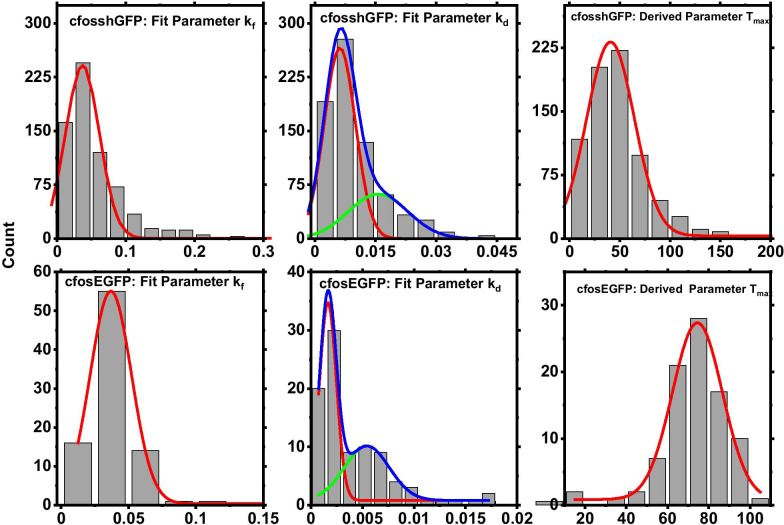
Frequency histogram of fit parameters: k_f_, and k_d_ along with the derived parameter T_max_. **a** Histograms of the formation rate constant k_f_ fits (solid red line) to Gaussian distribution with a mean of 0.0369 min^−1^. **b** Top row shows parameter distribution plots for cfos-shGFP transgenic mice (n =  ~ 700 cells). Bins with counts > 10 were considered for fit. We see a bimodal distribution of k_d_ values in cfos-shGFP mice indicating populations of neurons with different decay kinetics. Bottom row shows the corresponding plots for cfos-eGFP transgenic mice (n =  ~ 90 cells)

The ROIs of successful fits and their fit parameters (describing the rate of formation (k_f_), decay (k_d_) and extent of activation (A_0_)) are used for data analysis. We obtain the histogram of these rate constants and fit to normal distribution to estimate the mean values of these rate constants as described in Fig. 4. In case of decay constant k_d_ we see a bimodal distribution (verified through Akaike's Information Criteria (AIC), [1]) and we report both the decay constants in Table 3.

**Table 3:** Table summarizing the T_max_, k_f_ and k_d_ values from Gauss fit (in Fig. 4) to the respective distributions (Adj-R-Sq > 0.95)

Transgenic mouse	Tmax (min)	k_f_ (min^−1^)	k_d _(min^−1^)	
cfos-shGFP	41 ± 1	0.0369 ± 4 E−4	0.0062 ± 1E−4	0.016 ± 0.002
cfos-EGFP	74.5 ± 0.75	0.0369 ± 2E−4	0.002 ± 1E−4	0.005 ± 7E−4

From these values, we estimate the rise time and decay of the cfos-eGFP fluorescence following seizure or behaviour induced activation to be 1/k_f_ = 27 ± 3 min and 1/k_d_ = 200 ± 28 min (taking the faster component from Table 3), respectively. We use these rise and decay times to arrive at the sampling interval for further experiments. Similarly, cfos-shGFP expression following context exposure yields a rise and decay time of 1/k_f_ = 27 min and 1/k_d_ = 16 min (taking the faster component from Table 3), respectively. The faster component of decay constant is an indicator of how quickly the generated protein degrades. As expected, we see that the rise kinetics are identical for both these constructs considering the fluorophore is expressed under a cfos promoter.

**Fig. 5:**
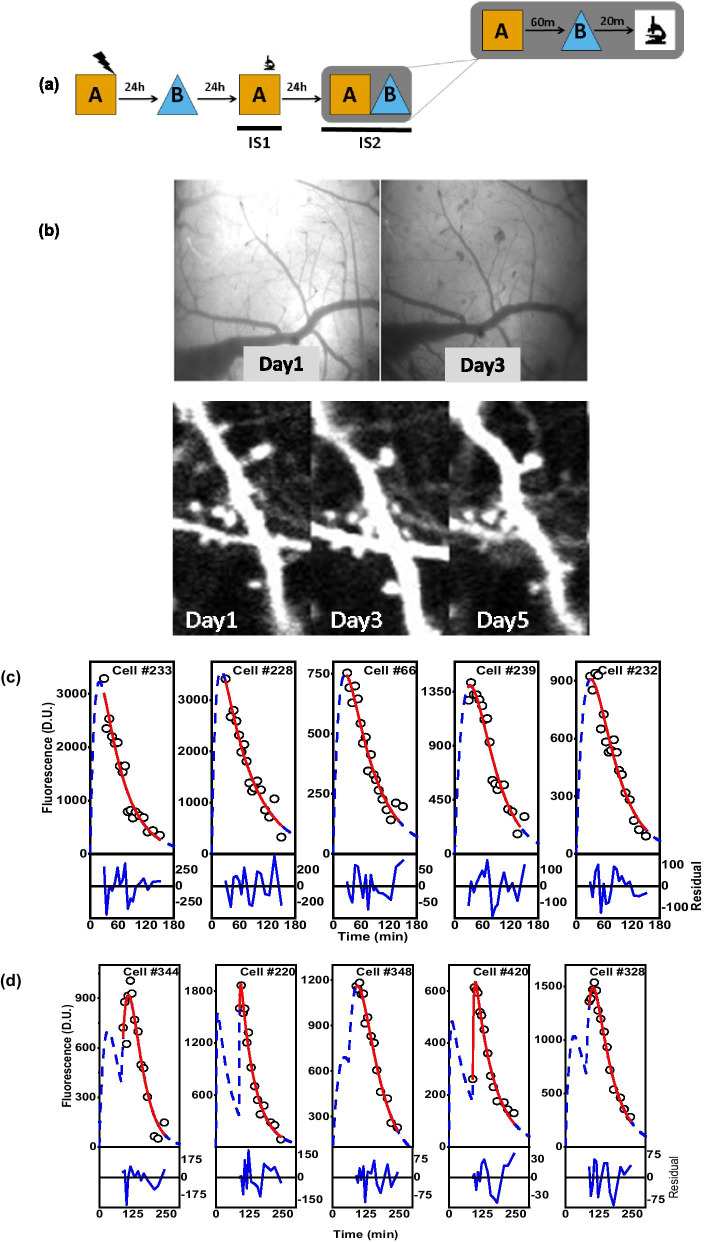

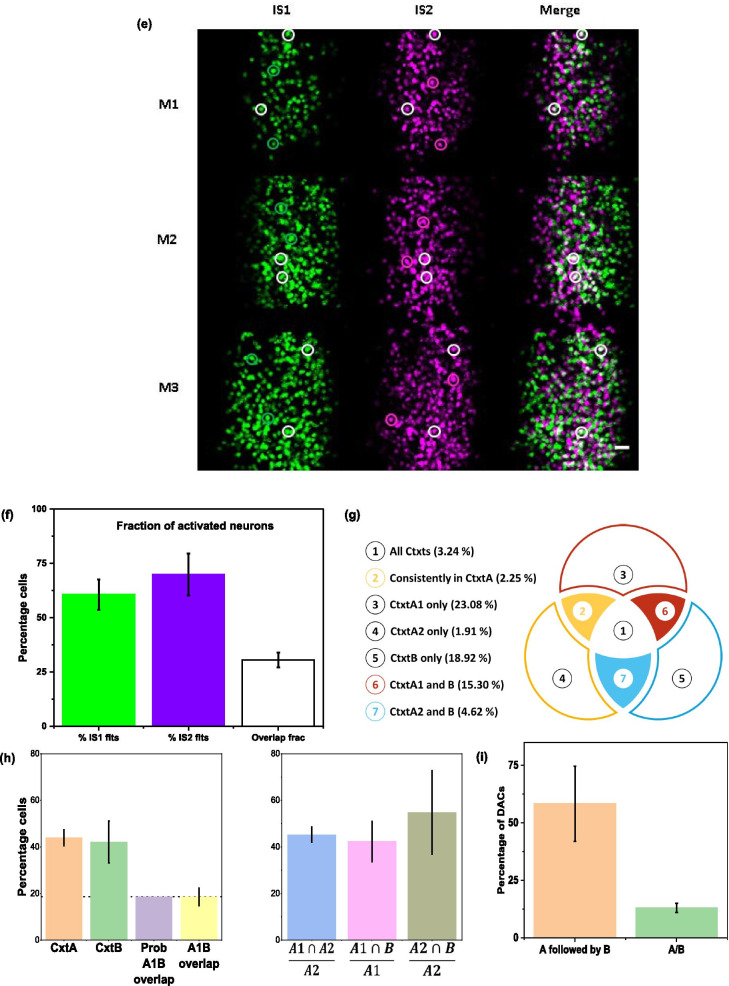
Identification and segregation of neuronal ensembles based on cfos protein temporal expression dynamics. **a** Schematic of behavioural paradigm used. **b** Longitudinal in vivo imaging of the same area across multiple days is achieved by using the unique blood vasculature of craniotomized mice. The representative images show the precision with which dendrites and their spines can be imaged across multiple days (1–5 days) in Thy1-YFP transgenic mice. The same method was followed for longitudinal in vivo imaging of cfos transgenic mice. **c** Representative plots of expression profile of single activation neurons. **d** Representative plots of expression profile of double activation neurons. The response of the neurons(DAC) fit (red line) is explained better by DAC profile Eq. 2 rather than SAC profile Eq. 1. The dashed blue line shows the fit spanning the entire laboratory time frame of t = 0 min where the mouse was exposed to the first context A, followed by t = 60 min where the mouse was exposed to context B. **e** Representative snapshot of cfos neuronal ensembles at ~ 90 min on imaging sessions IS1 (green) and IS2 (magenta) with overlapping ensembles shown in white. We constructed these images from snapshots of the RSc at ~ 90 min after the first context exposure of the imaging session. **f** Fraction of activated neurons on days IS1, IS2 and their overlap. **g** Visual schematic representing different categories of cells representing context information. The circles in the diagram represent the cellular ensemble that got activated in IS1(red), IS2(yellow) and IS2-60 min(blue). **h** Fraction of activated neurons on different retrieval events. Different bars on the left represent activated neurons (SAC or DAC) in response to different ctxt exposure—Orange: CtxtA on IS1, Green: CtxtB on IS2, Purple: chance factor determined as a product of CtxtA and CtxtB fractional activation., Yellow: observed overlap of CtxtA IS1 and CtxtB IS2 activation. The bars on the right side show the fractional activation to determine the overlap among/across the context with respect to CtxtA exposure in IS2. The blue bar represents the fraction of the cells that are part of CtxtA IS2 and are also part of CtxtA IS1. We estimate this fraction as this is independent of the activation across days. The pink bar represents the fraction of cells that are activated in CtxtA IS1and CtxtB IS2, while the grey bar represents the fraction of CtxtA IS2 cells that are active during CtxtB IS2. **i** The fraction of double activation cells in response to CtxtA followed by CtxtB on IS2 is significantly more than the fraction of double activation cells in response to single context exposure event

During this process, we observe that some of the cells have overlapping k_f_ and k_d_ values with high interdependency. We believe this is due to a difference in start time (when the cell got activated), resulting in a lower density of data points for a reliable estimate of the k_f_, rather than the lack of fit. We include some of these cell responses in Figs. 3c, 5c and d (Please see footnote of Tables 2, 4, 5).

**Table 4:** Summary of fit parameters of cfos-shGFP expression in response to dual context exposure data fit to Eq. 1

Fig No	Cell No	Amplitude (D.U.)	Error in Amplitude	k_f_ (min^−1^)	Error in k_f_	K_d_ (min^−1^)	Error in k_d_	Adj-R-sq	AIC
Figure 5c	Cell #233	4894	420.98677	0.09777	0.05748	0.02089	0.00332	0.9343	202.68672
Figure 5c	Cell #228	4856	282.65906	0.11699	0.07415	0.01537	0.00164	0.94395	187.96317
Figure 5c	Cell #66	1164	107.45433	0.07322	0.02521	0.017	0.00255	0.9444	146.82517
Figure 5c	Cell #239*	3807	261.832	0.03036	-	0.03037	0.00229	0.94465	174.71616
Figure 5c	Cell #232	1802	492.2815	0.04495	0.0202	0.02197	0.00738	0.9378	159.5796

^*^The kf and kd values for these cells were shared

**Table 5:** Summary of fit parameters of cfos-shGFP expression in response to dual context exposure data fit to Eq. 2

Fig No	Cell No	Amplitude (D.U.)	Error in Amplitude	k_f_ (min^−1^)	Error in k_f_	k_d_ (min^−1^)	Error in k_d_	t_d_	Error in t_d_	Adj-R-sq	AIC
Figure 5d	Cell #344*	1910	391	0.0306	-	0.0306	0.0066	82.12186	2.90802	0.88918	116.25422
Figure 5d	Cell #220	1777	131	0.45904	0.71656	0.0186	0.0016	86.19771	3.8883	0.97181	141.89924
Figure 5d	Cell #348*	1501	130	0.02756	-	0.01692	0.00191	60.3284	6.61903	0.97706	124.81833
Figure 5d	Cell #420	564	28	0.27809	0.06721	0.01378	9.17E-04	88.38919	0.2538	0.97612	106.37056
Figure 5d	Cell # 328	1689	194	0.05502	0.01922	0.01523	0.00208	76.87335	3.09424	0.98325	126.67097

*The kf and kd values for these cells were shared

Thus, we find the time to maximal activation (setting first derivative to zero) estimated from Eq. 1 using the mean k_f_ and k_d_ values to be different for cfos-eGFP (74.5 ± 0.7 min) and -shGFP mice (41 ± 1 min); in accordance with the genetic makeup of the mice and the properties of the transgene. In cfos-eGFP, the transgene is the fusion of eGFP and cfos protein, while in the cfos-shGFP just the GFP protein is expressed under cfos promoter. We note that these estimates of time to peak are different than what has been estimated from conventional studies. However, these estimates are not comparable to our estimates as further elaborated in the discussion section. Given the good fit of the cfos activation data, next we ask if we could use such a model to predict expression of cfos that occurs during memory formation, thereby enabling identification of neuronal ensemble that took part in representation of memory.

### Identification and segregation of neuronal ensemble following dual context exposure

In our efforts to identify and segregate the neurons that get activated in response to multiple events, we image the neuronal ensembles that respond to contextual fear conditioning. We use the following behavioural schematic (Fig. 5a) to train and image the contextual representation in cfos-shGFP transgenic mice. Briefly, the mice are trained to associate a foot shock in context A followed by safety training in context B the next day. Imaging sessions are carried out following retrieval tests that are carried out 24 h after safety training in context B.

In order to image the same region of brain and hence the cellular ensembles across the imaging sessions spanning days, we capture and utilise the vasculature of the brain as shown in Fig. 5b. This allows us to image the same region within ~ 0.5 mm (our field of view). Next, we utilise the system of co-ordinates (and co-ordinate transform as required) to further refine our positioning within the field of view. We find that these measures are sufficient to provide a localisation accuracy to locate not just the cell body but the same spines (Fig. 5b). We note that the positioning accuracy required for our purpose is at the cellular scale.

Our behavioural scheme consists of three context exposure sessions spread across two days. The mice are trained in CtxtA (shock context) and CtxtB (safe context). When tested for the fear memory they show significant freezing compared to baseline (Additional file 6: Fig. S6). Imaging is performed in two sessions during the retrieval days with first imaging session after exposure to context A. 24 h later, we image the mice following exposures to context A followed by context B. The exposures to contexts are 60 min apart. Thus, there are two imaging sessions: one following exposure to context A (IS1) on the first day of retrieval, and another after exposure to context B on the second day of retrieval (IS2). As described in the schematic, the second day retrieval consists of exposures to two contexts A and B separated by 60 min. In IS1, the imaging session consists of 20–180 min whereas in IS2 the imaging points consists of 90–300 min, at intervals of ~ 10 min. Since there are ~ 2500 ROIs to be processed, analysed, and classified, we develop a software-based workflow as described in “Materials and methods” (Additional file 1: Fig. S1).

In both these imaging sessions, we see two kinds of cellular responses: cells that are activated (I) once (single activation cell, SAC) and (II) twice (double activation cell, DAC) in response to context exposure. Figure 5c and d show representative cfos expression of SAC and DAC fit to Eqs. 1 and 2, respectively. The quality of the fit can be assessed through the residuals displayed at the bottom panel of each response. Tables 4 and 5 summarise the fit parameters. Due to their difference in initial slope and curvature around the peak of DAC compared to SAC, DAC fit the double activation cell profile corresponding to Eq. 2 better as determined by our criteria (AIC and Adj. R Sq ≥ 0.5). Additional file 4: Figure S4 illustrates this behaviour using an example cell response. The cell responses from second imaging sessions are fit twice, once with a delay of 60 min corresponding to time measurement starting from context B exposure (“delayed IS2”) and other with zero delay corresponding to time measurement starting from context A exposure. The investigated ROIs fit either Eq. 1 or 2 on at least one of the imaging sessions for us to consider it as a neuron. We find that 71 ± 6% of the investigated ROIs show a fluorescence response that fit at least in one of the imaging sessions.

Figure 5e shows the snapshot of the cellular activity at the RSc of cfos-shGFP transgenic mice. The green cells are the activated cells on imaging session 1 and the magenta are the activated cells on imaging session 2. The third panel overlays the cells that are activated on IS1 (green) and IS2 (magenta) to show the overlapping cells as white nuclei. Apart from these cells, as can be clearly seen from the images, there are cells that are activated in IS1 or IS2 only. These images represent the fraction of overlapped cells across two context exposures and this fraction is not known so far in RSc. We estimate this number to be ~ 30% (Fig. 5f white bar) and it is similar to what is observed in the other regions of the brain [30, 36].

### Retrosplenial cortex ensembles in response to dual context exposure

Next, we address whether there is a differential representation of a context when a new context is introduced. In the behavioural scheme (Fig. 5a) used to train and image the contextual representation in cfos-GFP transgenic mice, the mice are subjected to context exposure three times. We are interested in observing the neurons activated in response to these exposures or experiences. A neuron can get activated in response to anyone or two or all of these experiences. Hence, we use our method to segregate the activated neurons into different categories as described below.

Based on the temporal profile of expression, we interpret the SAC that are identified following an exposure to a given context as representing that context or experience only, whereas the DAC as representing two events e.g., representing two context exposures or context exposure followed by a spontaneous event or a spontaneous event followed by a context exposure. Next, we compare these groups with our behavioural scheme to reason and assign these different classes to the corresponding memory representations, e.g., since IS1 follows retrieval event in context A and SAC IS1 responses are consistent with Eq. 1 representing single events, we assign them to context A cellular ensemble. Similarly, these SAC and DAC are assigned to one of the eight categories as listed in Table 6. The Venn diagram in Fig. 5g represents the various categories of cells representing a context and their expected session-wise fit result(s). We describe below the categories and their rationale.

**Table 6:** Based on cellular response profile and fits as SAC or DAC in imaging sessions IS1 and IS2, the ROIs are classified into one of the eight categories as represented in the Venn Diagram (Fig. 5g)

Venn Diagram section	IS1 (Ctxt A1)	IS2 (Ctxt A2)	IS2 (CtxtB)	Physiological meaning
1	1	1	1	Cell that is present in all contexts
2	1	1	0	Cell that is present in CtxtA only
3	1	0	0	Cells present in IS1 CtxtA only
4	0	1	0	Cells present in IS2 CtxtA only
5	0	0	1	Cells present in B only
6	1	0	1	Cells present in IS1 CtxtA and IS2 CtxtB
7	0	1	1	Cells present in IS1 CtxtA and IS2 CtxtB
8	0	0	0	Cells not activated in all contexts

One subset (Category (1)) of cells that are activated in response to all three experiences, i.e., these cells show a profile that is consistent with SAC IS1 (CtxtA) and DAC IS2 (CtxtA, CtxtB).

Three categories describe the subsets of cells activated to a single experience only, namely categories (3), (4), (5). These cells have expression profiles corresponding to one of the following: SAC in IS1 (CtxtA), SAC in IS2 (CtxtA), and SAC IS2 with 60 min delay (CtxtB) respectively.

Three other categories (Categories 2, 6 and 7) represent the subset of cells that are reactivated. Category (2): These are cells that consistently respond only to context A i.e., SAC in IS1 (CtxtA) and SAC in IS2 (CtxtA). Category (6) are cells activated in IS1and fit a SAC or DAC profile and then reactivated in IS2 with a profile that can be classified to DAC or a SAC with 60 min delay. We interpret these cells as common between CtxtA IS1 and CtxtB. Similarly, category (7) are cells, activated in response to CtxtA IS2 and CtxtB, i.e. DAC in IS2.

Finally, category (8) are the ROIs/cells that are not activated in response to any of the experiences as both IS1 and IS2 responses did not fit, i.e., the ROIs did not fit as a SAC or DAC.

Following this classification system, we estimate the fraction of neurons activated in response to single context exposure(CtxtA) during the first retrieval event as 44 ± 3% (Fig. 5h, orange bar) and the fraction of cells that are activated in CtxtB, i.e., category 6, as ~ 42 ± 9% (Fig. 5h, green bar). This allowed us to ask if the ensembles representing A and B are unique. We test the uniqueness of these ensembles by estimating the fraction of the cells that are common between these two ensembles. We reason that an above chance overlap would indicate that the same population is getting activated in both the contexts, while an at chance overlap would indicate that the neurons recruited during the corresponding context retrieval are different and independent. We find the overlap fraction to be at chance (Fig. 5h, yellow bar, 19 ± 4%) suggesting that the population representation of these contexts is indeed unique and independent.

Next, we proceed to investigate if it is possible to link two distinct memory representations by retrieving these memories close in time. It is interesting to note that previously it has been shown in hippocampus, that if the memory formation were to happen within a time window, then their likelihood of overlapped neuronal representation is high [7, 33]. We ask if such overlap can be seen in Rsc following temporally close context retrieval as opposed to acquisition. We do this by estimating the chance of a neuron getting activated in CtxtA in IS1 (first exposure of context A) and chance of it getting activated in CtxtB (Fig. 5h, pink bar, 42 ± 8%). We then compare it with the chance of a neuron getting activated in CtxtA in IS2 and CtxtB simultaneously (Fig. 5h, brown bar, 55 ± 18%). Since CtxtA during IS2 and B are occurring closer in time (60-min fraction) than CtxtA in IS1 (24-h fraction), if such temporal linkage were to occur, we would see the 60-min fraction being greater than 24-h fraction. However, we find both of them are comparable despite a trend of 60 min fraction being higher than the 24 h fraction (Fig. 5h). Additionally, we find these ratios comparable to that of reactivation probability estimated as fraction of neurons from IS2 CtxtA that got activated during CtxtA IS1.

Next, we proceed to test if our method can identify double activated cells even in this scenario. We estimate the fraction of DAC in ctxtA during IS2 and compare it with DAC identified during IS1 or delayed IS2. Our comparison reveals that the fraction of DAC in CtxtA during IS2 is indeed significantly higher than DAC in IS1 or delayed IS2 (Fig. 5i). We see DAC of context A followed by B (58.3 ± 16.3%) to be significantly different (t test: p < 0.002, one tailed) than DACs of either context A or B (~ 13 ± 2%), thus establishing the fact that following a protein expression kinetics allows one to segregate cells even when the mouse is exposed to two contexts close in time.

**Fig. 6:**
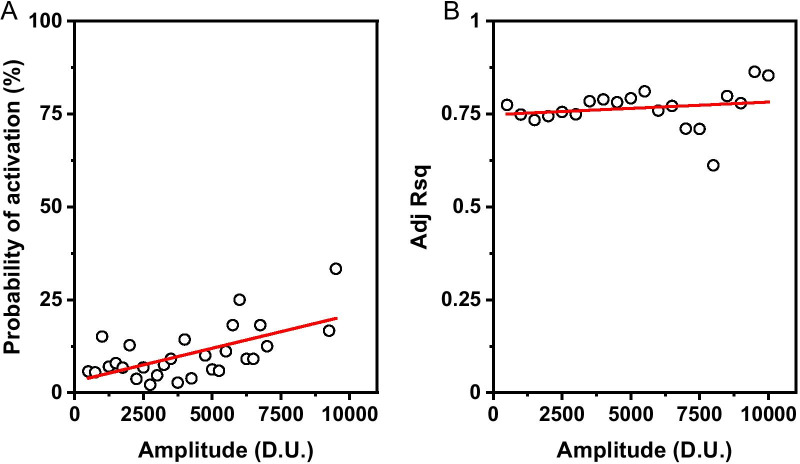
**a** Correlation between the amplitude of activated neurons in IS1 and the probability of reactivation of the same neurons in IS2. The probability of reactivation is calculated as N_fit_/N_bin_ where N_fit_is the fraction of neurons that fit with adj R-sq greater than 0.5, N_bin_is the total number of cells within the amplitude bin I_bin_. (Bin size = 500). **b** Correlation between the amplitude of fit neurons with the adj R-sq

Since cfos is an IEG immediately downstream of CREB, we ask, could the levels of cfos be predictive of reactivation probability during repeated retrieval? We estimate the reactivation probability by binning the neurons according to their amplitudes and estimating the fraction of fit neurons in each of these bins. The ratio between the number of fit or activated neurons to the total number of neurons represents the fraction of reactivated neurons in each intensity bin. We define this ratio as the probability of reactivation in that bin. Figure 6A shows this reactivation probability as a function of amplitude of cfos signal. We see a weak, but a significant correlation (R > 0.6) exists between the amplitude and the reactivation probability. In order to rule out the possibility that the difference in amplitude could bias the R.Sq of the cellular response fit and hence the reactivation probability, we also plot the Adj. R.Sq of the fits as a function of amplitude in Fig. 6B. We see that Adj. R Sq is invariant with respect to the amplitude of the cfos signal as indicated by a near zero slope of 3E-6 ± 4E−6.

## Discussion

In summary, we can follow the IEG protein expression dynamics to identify and segregate the activation of neurons into different groups as per the behavioural events. Conventionally, three probes are required to mark three retrieval events; however, our method requires one IEG-fluorophore to mark three retrieval events. Since our method utilises the temporal profile of IEG expression, as opposed to intensity threshold, for identification, segregation, and classification of neurons, we are able to increase the specificity with which we identify behaviourally relevant neuronal ensembles.

Interestingly, we find cfos protein's maximum expression takes place around ~ 74 min for fos-eGFP transgenic mice, and ~ 40 min for fos-shGFP transgenic mice. These differences are consistent with shGFP having a shorter half-life.

Previous studies report cfos expression peaks between 90 and 120 min for protein as detected by immunocytochemistry (IHC) analysis [6, 26]. While we measure the cfos fluorescence in these mice and thus report the peak protein expression directly, other methods measure peak time to obtain maximum number of positive cell counts. The time to peak, in such cases represent the time at which maximum number of cells reach above threshold fluorescence. This is not necessarily the time at which cfos expression in a given neuron reaches a maximum. Since the IHC is a single point measurement in time, it lacks the ability to distinguish if the cell is in the rising or falling phase of the expression. Thus, a variation in the onset time, which is hard to control in IHC as the measurements are compared across different mice, can render cells in different phases with same fluorescence. Given the fact that the positive cells are identified using thresholds, their measurement yields an apparent peak for cell counts and the time at which this peak occurs is different from the time to peak expression of the protein itself. On the other hand, our method directly measures the rise and decay of the cfos expression from single cells through fluorescence from individual mice. Being a single cell measurement arising from same mice, limits, if not eliminates, the contribution of onset time variation. Thus, we argue that in vivo fluorescence signal is more sensitive and a direct measure of cfos-eGFP protein concentration level in a neuron in real time, compared to immunocytochemistry, leading to the discrepancy in reported time range of maximal cfos protein expression. Thus, the other methods measure the time to get maximal cell fraction [6, 16, 38] rather than the maximal expression of protein in the cell.

Estimates from our method allowed us to probe a dual context exposure interaction and show that RSc activation following different context exposures can be studied using our method.

We find the level of cellular activation elicited by CtxtA and CtxtB are nearly same and is similar to what has been found in other regions of the brain with dense encoding [36]. We see responses of ~ 20% (Data not shown) of neurons still show cellular response profile that enables them to be classified as active in context A even though the exposure has occurred 90 min prior to first imaging data point of imaging session 2. In order to arrive at an upper bound for fraction of cells that we could have missed we took the IS1 SAC data from 90 min onwards and asked what fraction of those cells would still qualify as SAC (Additional file 5: Fig. 5b). We estimate that even after 90 min we are still able to fit ~ 50% of cells. Interestingly, many of the neurons activated in context B show expression dynamics consistent with dual activation profile (Fig. 5i), we interpret this as a neuronal response to context A as well as context B exposure. Though, the activated cellular fraction at RSc has not been reported before, our estimate is comparable to the ensemble size typically obtained in other regions through conventional studies [18].

Further we assign the SAC from context B exposure and DAC from context A exposure of IS2 to context B cellular representation. We note this fraction ~ 45% for context B is similar to that of context A cellular fraction. Thus, consistent with our hypothesis, these two contextual representations can be identified and assigned to different cell populations using cellular activation profiles.

One of the defining events of IS2 is the arrival of the context B exposure after context A. It is known that contextual exposure could be temporally linked through cellular activation [7, 33]. This being the case, we expect a larger fraction of activated cells following context A exposure in IS2 should be DACs. Thus, we compare the fraction of DAC seen in response to context B following context A exposure (Fig. 5i, orange bar) with that of DAC seen in response to a single context A or B (Fig. 5i, green bar). Such a piece of information is hard to obtain through conventional molecular probing methods since the comparisons are made at a population level instead of a single cell.

One of the confounding factors in conventional method is the difficulty in assigning the cell response to behavioural event. For example, DAC in response to single context exposure, either in context A or context B, would be falsely identified as representing two contexts in conventional methods. More importantly, majority of the ROIs that did not fit either single or double activation, would have been classified as representing one of the contexts when in fact they do not represent IEG activation intended through behavioural exposure. While it is possible, the lack of fit could represent a different model of activation profile, we see a vast fraction of the non-fit ROIs to be invariant with respect to time. Since their fluorescence is measured to be above the baseline, these cells might add noise in the conventional estimates of cell fractions.

Further, we could identify the activated ensembles following different contextual memory retrieval in RSc as early as 24 h. Taking advantage of these, we probed if distinct memories once formed could be linked through closely spaced retrieval events. As expected, we find that mere retrieval of two distinct memories close in time is not sufficient to link the RSc ensembles representing these memories if the memories themselves are acquired 24 h apart.

## Materials and methods

### Transgenic mice

cfos-eGFP (B6.Cg-Tg(Fos/EGFP)1-3Brth/J Stock no: 014135) and cfos-shGFP (B6.Cg-Tg(Fos-tTA,Fos-EGFP*)1Mmay/J Stock no: 018306) transgenic mice were obtained from Jackson Laboratory, USA and maintained at the Central Animal Facility, IISc. All protocols were approved by the Institute Animal Ethics Committee.

### Craniotomy

Transgenic mice underwent a craniotomy to enable in vivo imaging [34]. A sterile 6 mm coverglass was positioned over the skull between the bregma and lambda, centred at retrosplenial cortex (RSc). The coordinates of the imaging area (RSc: 2 mm from Bregma, 0.5 mm laterally) were arrived at by visualising the blood vasculature. The mice were anesthetised using a solution of fentanyl (0.05 mg/kg), midazolam (5 mg/kg), and medetomidin (0.5 mg/kg) dissolved in saline.

### Artificially and behaviourally induced IEG expression

Artificial IEG induction was produced by injecting mouse with 2 mg/kg bicuculline intraperitonially to induce a seizure. Mild seizure symptoms were observed. On completion of the seizure, mouse was anesthetised with FMM to proceed to in vivo imaging. We injected one cfos-eGFP mouse for this experiment.

Behaviour paradigm: Mice were trained to associate a mild foot-shock (0.7 mA, 2 s) in context A (70% ethanol, spaced grill floor) on training day (2 min 30 s, Day 1). The next day, these mice were placed in context B (20% ethyl acetate, smooth floor, triangle chamber feature) without shock for 2 min 30 s (safety context training). After 24 h (Day 3), mice were placed again in context A to test for memory recall. On Day 4, mice were placed in context A followed by context B separated by a time period of 60 min to test their ability to discriminate similar contexts at a recent time (24/48 h). We used four cfos-shEGFP mice for this experiment followed by imaging. Of the four, one of the mice did not learn (no freezing in any of the retrievals).

### Imaging setup

In vivo imaging was performed on a custom-built two-photon setup based on a Zeiss upright microscope (AxioExaminer Z1) equipped with a 25 × water immersion objective (NA 1.05, WD 2 mm, Olympus XLPLN25XWMP2). Femtosecond pulses from an ultrafast Ti:Sapphire laser (Newport, Tsunami) whose intensity was modulated using a half-wave plate (Thorlabs, AHWP05M) and a polarizer (Thorlabs, GL10-B) is used as light source. The excitation beam is raster scanned using a galvo scanning mirror (Thorlabs, GVSM002) before entering the microscope body and is focussed on the imaging plane using the objective lens. The fluorescence that is collected by the objective lens in an epi-illumination geometry is then separated using a dichroic before being detected by photomultiplier module (H7422, Hamamatsu Corporation, Japan).

A low noise current preamplifier (Stanford Research Systems, SR570) was used to amplify the photomultiplier tube photocurrent, which was further digitized using a data acquisition board (National Instruments, PCI-6110). ScanImage (r 3.8.1) software was used to interface instrument control and generation of galvometric scan command. Image acquisition was accomplished using a custom Matlab script interfaced with z-drive of the microscope. The digitized signal was analysed using Matlab, Origin and ImageJ for further analysis.

### Estimation of fluorescence from cfos expression neurons

The in vivo images were analysed manually using a modified version of Time Series Analyzer plugin (Time Series Analyzer Plugin, Balaji 2014, https://imagej.nih.gov/ij/plugins/time-series.html) in ImageJ to quantify the fluorescence signal. The modified version of the plugin is publicly available as Java Repository inGitHub (GitHub link: https://github.com/TheNeurodynamicsLab/ImageJ_NDLPlugins). Additional file 1: Fig. S1 describes the steps to extract the fluorescence signal from each neuron to obtain the fluorescence value of a neuron at a given time point. Briefly, for each individual neuron, the peak intensity at given time point was quantified by manually selecting the nucleus as the region of interest (ROI). The mean pixel intensity of the ROI through each z-stack was obtained and fit to a Gaussian function to estimate the activity of the ROI at a particular time point (Additional file 1: Fig. S1). We are able to identify 2527 neuronal ROIs from 5 mice (3 cfos-shGFP, 2 cfos-eGFP).

### Classification of SAC and DAC through curve fitting

Curve fitting analysis of fluorescence as a function of time for each ROI was done in Origin(v2020b)’s user-defined NLFit function using Levenberg–Marquardt algorithm. The parameters were set as follows for data fitting to Eq. 1: Parameter A was initialised to the maximum fluorescence of the ROI observed in the imaging session, while rate constants k_f_ and k_d_ are initialised to 0.01 and 0.001, respectively, at the start of the Levenberg–Marquardt algorithm for least squares minimisation. For data fitting to Eq. 2, the additional parameter t_d_ was initialised with a value of 60 min. Post data fitting to Eqs. 4 and 5, the preferred model (SAC or DAC) was selected based on Akaike Information Criterion (AIC). In brief, AIC measures the information loss incurred in choosing a fit model given the observed data and degrees of freedom. Thus, it considers the difference in number of parameters used in a fit as well as the goodness of the fit. A model with low AIC explains the observed data with minimal loss of information without over fitting, and hence, is preferred. The goodness of fit was determined by an Adj. R Sq value and it was set to be greater than 0.5 to identify the selected ROI as an activated cell of the preferred model.

### Supplementary Information

**Additional file 1: Fig. S1.** Extraction of fluorescence values. (a) Workflow describing the steps for data extraction. (b) Left: Snapshot of field of view at 90 mins. Yellow circle and inset top represent one neuron. Top inset: Optical sections of the neuron at different Z positions in a stack. Bottom: Gaussian fit to obtain mean pixel intensity or fluorescence of a neuron at a given time point. The open circles represent the mean pixel intensity of the neuron at a slice/Z position. The red line represents the Gaussian fitting of mean pixel intensity as a function of Z position.

**Additional file 2: Fig. S2.** Left image is an optical section of cfos-shGFP at 90 mins, Right image shows the ROIs that are identified, centred and their background cleared.

**Additional file 3: Fig. S3.** Seizure induced expression of IEG coupled fluorescence is described by first order consecutive kinetics: (a) Schematic of IEG induction to bicuculline administration and following its dynamics in an anesthetised transgenic mouse through in-vivo imaging of the retrosplenial cortex (RSc). (b) Select regions of interest centred around cells #39, #41, #46 and #5, are arranged as time series show the change in fluorescence across the entire cell nuclei. (c) Quantitative measure of cellular response extracted from the time series images through custom built software for four representative cells are shown as open circles. The open circles are obtained using the workflow (SFig. 1) and represent the activity of a neuron at a given time. The red line is the fit of this activity to Eq. 1. Blue dotted line extends the solid red line to the activity of the cell outside of the imaging time frame as predicted by our model. See table 3 for fit parameter details.

**Additional file 4: Fig. S4.** Comparison of a DAC fit to Eq 1 with (t = 60min, solid green line) and without (t = 0 min, solid red line) delay along fit to Eq 2(solid blue line).

**Additional file 5: Fig. S5.** Fraction of IS1 SAC ROIs fit to equation 1 using fluorescence from imaging time points comparable to IS2 imaging time points (i.e. 80 min onwards from first context exposure) show that ~50% of data fit based on our criteria. (a) The graph shows the average responses during IS2 of “non-fit” ROIs from one mouse represented as open circles as function of time. Black solid squares are the corresponding values for IS1. The change in fluorescence over ~15 imaging sessions over 160 mins show a moderate decrease of about 20 – 30 % as compared to cellular profiles that show an order of magnitude increase from the baseline (~0 for fit cells). (b) The graph represents fraction of cells fit to equation 1 when the fit was performed with imaging time points comparable to IS2 imaging time points, i.e., ~80 min onwards. Orange bar is that fraction of fit ROIs of IS1 SAC but not IS2 SAC or DAC (mean of 3 mice, n = 32, 213, 162 ROIs) whereas green bar is the fraction fit ROIs of IS1 SAC (mean of 3 mice, n = 137, 327, 319 ROIs).

**Additional file 6: Fig. S6.** Percentage freezing for the mice used in dual exposure contextual fear conditioning paradigm. M1: Red circle, M2: Black square, M3: Blue triangle.

## Data Availability

The datasets used and/or analysed in the current study are available after publication from the corresponding author on written request.

## Appendix

### Theory

#### Analytical description of IEG expression dynamics

In order to model IEG expression kinetics in a neuron, we assume that a pool of mRNA is present which is translated to protein in response to behaviourally relevant neuronal activity or signal [15, 32]. In response to a signal, mRNA (A) is converted to protein (B) with a forward rate constant of protein formation (k_f_). One of the marked features of IEG proteins is their auto degradation resulting in transient expression. As the protein forms, ubiquitination degrades the protein, and we assume such a reaction to be first order in protein concentration with a degradation rate constant of k_d_. Thus, the IEG expression kinetics can be considered as consecutive first-order reactions (Fig. 1a).

$${\text{A}}\mathop{\longrightarrow}\limits^{{{\text{k}}_{{\text{f}}} }}{\text{B}}\mathop{\longrightarrow}\limits^{{{\text{k}}_{{\text{d}}} }}{\text{C}}$$

where A is the concentration of the substrate for protein synthesis, B represents the number of protein molecules and C is that of the degraded products.

Given the first order nature of the reaction we proceed to write the rate equations for A and B as follows,

$$\frac{d\left[ A \right]}{{dt}} = - \,k_{f} \left[ A \right]$$

$$\frac{d\left[ B \right]}{{dt}} = k_{f} \left[ A \right] - k_{d} \left[ B \right]$$

In order to solve for [B] the number of protein molecules that is going to be present transiently we need to solve the above coupled differential equations. We proceed by solving for B in Laplace space. Thus, using Laplace transform to transform the equations above we get,

$$\mathop \smallint \limits_{0}^{\infty } \frac{dA\left( t \right)}{{dt}}e^{ - st} dt = - k_{f} \tilde{A}$$

$$\mathop \smallint \limits_{0}^{\infty } \frac{dB\left( t \right)}{{dt}}e^{ - st} dt = k_{f} \tilde{A} - k_{d} \tilde{B}$$

which can be reduced to the below equations,

3 $$- A\left( 0 \right) + s\tilde{A}\left( s \right) = - k_{f}$$

4 $$- \,B\left( 0 \right)\, + \,s\tilde{B}\left( s \right)\,\, = \,k_{f} \tilde{A}\left( s \right)\, - \,k_{d} \tilde{B}\left( s \right)$$

where A(0) is the maximum substrate concentration per signalling event. B(0) is the concentration of B at time t = 0, hence B(0) = B_0_. Rearranging Eq. (3), we get the value of A(s).

$$\tilde{A}\left( {\text{s}} \right) = \frac{{{\text{A}}\left( 0 \right)}}{{{\text{s}} + {\text{k}}_{{\text{f}}} }}$$

Using this in Eq. (4) and rearranging for ~ B(s), we get,

5 $$\tilde{B}\left( s \right) = \frac{{A\left( 0 \right){\text{k}}_{{\text{f}}} }}{{\left( {s + k_{f} } \right)\left( {s + k_{d} } \right)}} + \frac{B\left( 0 \right)}{{\left( {s + k_{d} } \right)}}$$

At this stage we have several possibilities for B(0) each one of them corresponding to different and unique physical scenarios as discussed in special cases.

Equation (5) represents the number of IEG molecules in Laplace space. Using partial fractions and obtaining the inverse Laplace transform, we have,

$${\text{B}}\left( t \right) = \frac{{A_{o} k_{f} }}{{k_{f} - k_{d} }}\left( {e^{{ - k_{d} t}} - e^{{ - k_{f} t}} } \right) + B\left( 0 \right)e^{{ - k_{d} t}}$$

A fluorescence signal from a sample (F(t)) is proportional to the number of molecules (B(t)). Using the quantum efficiency (ϕ_f_), the absorption cross-section of the fluorophore (∈_A_) and the collection efficiency (C_f_), we can write,

$$F\left( t \right) = B\left( t \right)\phi_{f} \in_{A} C_{f}$$

For a given imaging system, the fluorescence signal (F(t)) is directly proportional to the number of molecules present at any given time (B(t)).

Thus, the equation can be rewritten as,

Eq. 1 in Fig. 1b $$\begin{aligned} {\text{F}}\left( t \right) & = \left[ {\frac{{A_{o} k_{f} }}{{k_{f} - k_{d} }}\left( {e^{{ - k_{d} t}} - e^{{ - k_{f} t}} } \right) + B\left( 0 \right)e^{{ - k_{d} t}} } \right] \\ {\text{F}}\left( t \right) & = \left[ {\frac{{A_{o} k_{f} }}{{k_{f} - k_{d} }}\left( {e^{{ - k_{d} t}} - e^{{ - k_{f} t}} } \right)} \right]\quad {\text{for B}}\left( 0 \right){\text{ is zero}}. \\ {\text{F}}\left( t \right) & = \left[ {\frac{{A_{o} k_{f} }}{{k_{f} - k_{d} }}\left( {\alpha e^{{ - k_{d} t}} - e^{{ - k_{f} t}} } \right)} \right]\quad {\text{for B}}\left( 0 \right){\text{ is a non - zero constant}}. \\ \end{aligned}$$

Using a similar approach, we can generalise the equation for multiple activation events in a neuron, as follows:

$$F_{{\text{n}}} \left( t \right) = \sum\limits_{{{\text{i}} = 1}}^{{\text{n}}} {F_{1} \left( {t - \left( {{\text{i}} - 1} \right)t_{d} } \right){\mathcal{H}}_{{\left( {{\text{i}} - 1} \right)t_{d} \left\{ {{ }\begin{array}{*{20}c} {0, t < t_{d} } \\ {1, t \ge t_{d} } \\ \end{array} } \right.}} }$$

In case of a double activation event, the equation simplifies to:

Eq. 2 in Fig. 1c $$F_{2} \left( t \right) = F_{1} \left( t \right) + F_{1} \left( {t - t_{d} } \right){\mathcal{H}}_{{t_{d} }} \left\{ \begin{gathered} 0,\quad t < t_{d} \hfill \\ 1,\quad t \ge t_{d} \hfill \\ \end{gathered} \right.$$

where t_d_ is the time of the second activation event or signal.

#### Special cases

At this point we consider some special cases where the following physically relevant scenarios present as modifications to our equation above:

Case (i): There is background non-zero steady state expression of cfos fluorescence is represented by B(0) = constant. We note that this requires the frequency of the stochastic activity such that their production and decomposition lead to a quasi-steady state. The corresponding final expression for this case is given by, ${\text{F}}\left( t \right) = \left[ {\frac{{A_{o} k_{f} }}{{k_{f} - k_{d} }}\left( {\alpha e^{{ - k_{d} t}} - e^{{ - k_{f} t}} } \right)} \right]$ where the co-efficient of the exponent representing the decay is modified but overall functional form largely remains unchanged.
Case (ii): Stochastic activity: First we note that stochastic activity induced expression and decay is not any different from event induced expression when the frequency of activation is sparse. (Case(i) represents where the frequency is high leading to a steady state expression). In these cases, the cell will fit to single activation or dual event activation profile with stochastic event response being one of the component.
Case (iii): Decay due to cellular decomposition /photo-bleaching: Such responses do not have a formation component. Our analysis in terms of classifying the cells based on activation profile does not make assumption about the nature of fluorescence decay. This could arise from cellular degradation or photo-bleaching or any other time dependent process. So, in principle our expression would cover any such decay as long as the defining parameters (such as incident intensity) are kept constant during the experiment. Thus, this would reflect as modified value for k_d_ but not affect or alter the functional form of the resulting equation. Further, it has been shown that the time scales (ms) [22] at which the photo-bleaching affects the fluorescence intensity is longer than the pixel dwell time (4 μs) that we have used for imaging. Additional file 5: Fig. S5 shows the response of non-responsive (did not fit) cells and as one can see the responses are more or less constant showing minimal reduction in intensity as compared to the cellular responses shown in the rest of the figures (Figs. 2, 3 and 5).

As it can be seen all these cases do not alter our final expression in terms of the functional form but do modify or alter the meaning of some of the parameters. Thus, a method that does not rely on the value of the kinetic parameters but utilises the functional form to test if the cellular response conforms to it (fit) would be able to classify the cells relatively free of confounds.

## Abbreviations

SAC: Single activation cell
DAC: Double activation cell
RSc: Retrosplenial cortex
shGFP: Short half-life Green fluorescent protein
IEG: Immediate early gene
Adj. R sq: Adjusted R-square
