# Supplementary figures and images for: In vivo imaging of immediate early gene expression dynamics segregates neuronal ensemble of memories of dual events
Source: Mol Brain. 2021 Jun 29;14:102. doi: 10.1186/s13041-021-00798-3 (PMC8243579; doi:10.1186/s13041-021-00798-3)

**S1:**


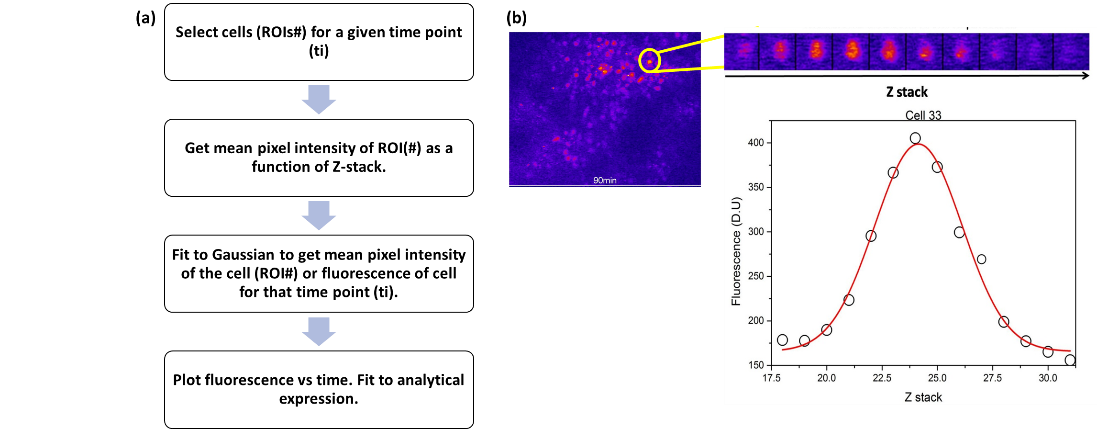

Supplement: Supplementary file 1 — Additional file 1: Fig. S1. Extraction of fluorescence values. (a) Workflow describing the steps for data extraction. (b) Left: Snapshot of field of view at 90 mins. Yellow circle and inset top represent one neuron. Top inset: Optical sections of the neuron at different Z positions in a stack. Bottom: Gaussian fit to obtain mean pixel intensity or fluorescence of a neuron at a given time point. The open circles represent the mean pixel intensity of the neuron at a slice/Z position. The red line represents the Gaussian fitting of mean pixel intensity as a function of Z position. [file 13041_2021_798_MOESM1_ESM.docx]

**S2:**


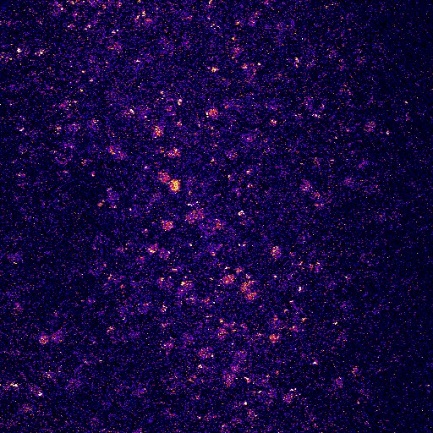

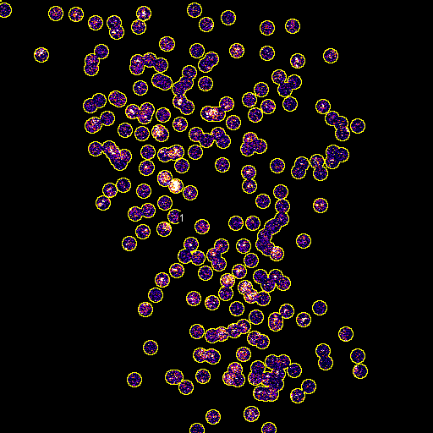

Supplement: Supplementary file 2 — Additional file 2: Fig. S2. Left image is an optical section of cfos-shGFP at 90 mins, Right image shows the ROIs that are identified, centred and their background cleared. [file 13041_2021_798_MOESM2_ESM.docx]

**S5**


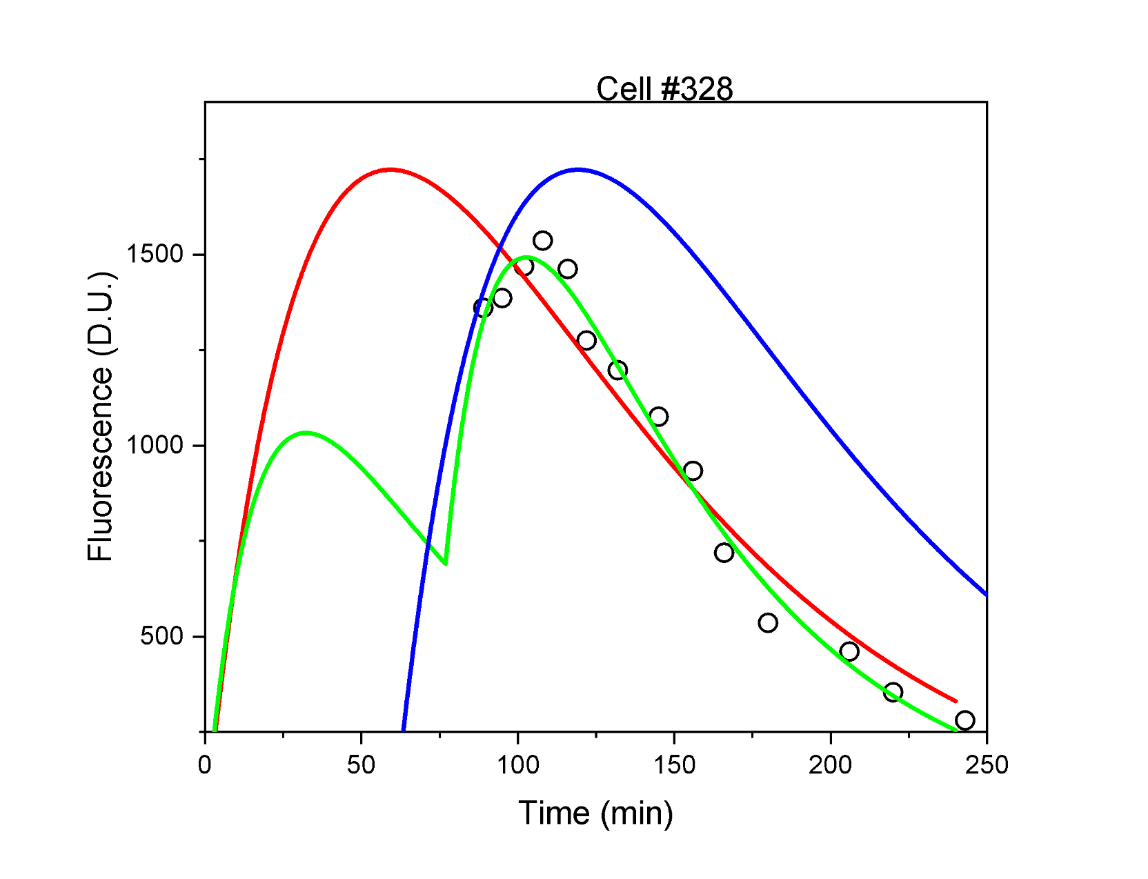

Supplement: Supplementary file 4 — Additional file 4: Fig. S4. Comparison of a DAC fit to Eq 1 with (t = 60min, solid green line) and without (t = 0 min, solid red line) delay along fit to Eq 2(solid blue line). [file 13041_2021_798_MOESM4_ESM.docx]

**S5:**

**(b)**


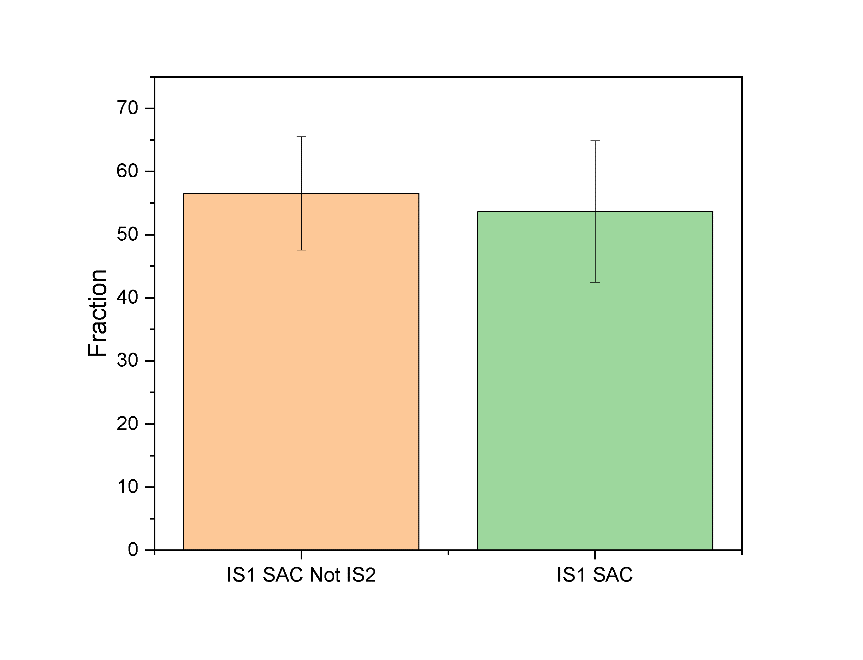


**(a)**


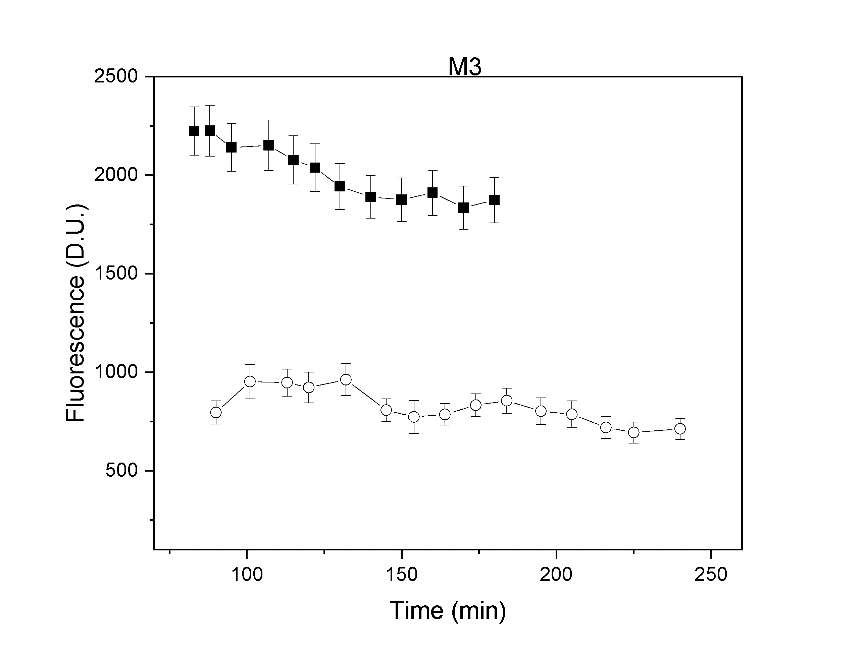

Supplement: Supplementary file 5 — Additional file 5: Fig. S5. Fraction of IS1 SAC ROIs fit to equation 1 using fluorescence from imaging time points comparable to IS2 imaging time points (i.e. 80 min onwards from first context exposure) show that ~50% of data fit based on our criteria. (a) The graph shows the average responses during IS2 of “non-fit” ROIs from one mouse represented as open circles as function of time. Black solid squares are the corresponding values for IS1. The change in fluorescence over ~15 imaging sessions over 160 mins show a moderate decrease of about 20 – 30 % as compared to cellular profiles that show an order of magnitude increase from the baseline (~0 for fit cells). (b) The graph represents fraction of cells fit to equation 1 when the fit was performed with imaging time points comparable to IS2 imaging time points, i.e., ~80 min onwards. Orange bar is that fraction of fit ROIs of IS1 SAC but not IS2 SAC or DAC (mean of 3 mice, n = 32, 213, 162 ROIs) whereas green bar is the fraction fit ROIs of IS1 SAC (mean of 3 mice, n = 137, 327, 319 ROIs). [file 13041_2021_798_MOESM5_ESM.docx]

**S6:**


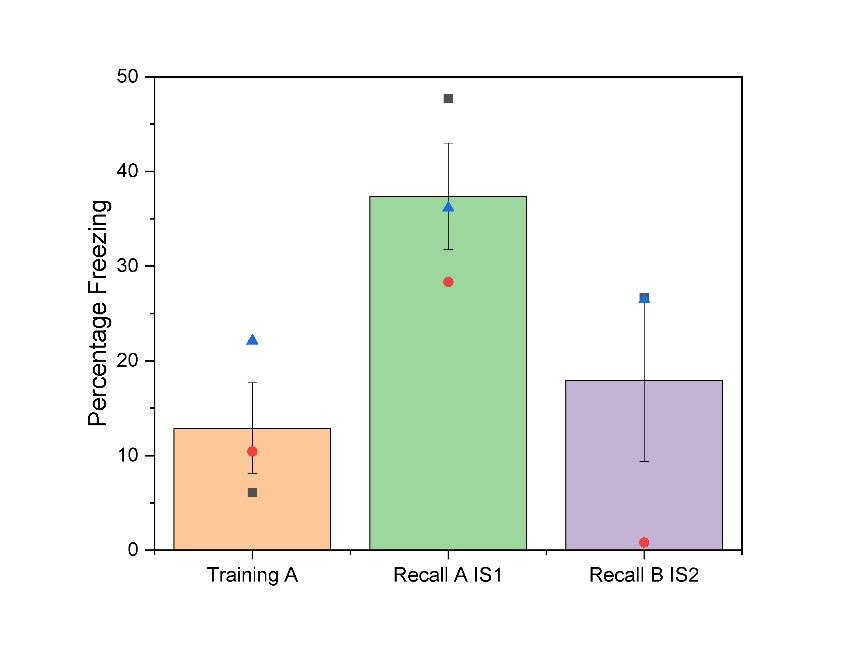

Supplement: Supplementary file 6 — Additional file 6: Fig. S6. Percentage freezing for the mice used in dual exposure contextual fear conditioning paradigm. M1: Red circle, M2: Black square, M3: Blue triangle. [file 13041_2021_798_MOESM6_ESM.docx]
