# Supplementary material for: In vivo imaging of immediate early gene expression dynamics segregates neuronal ensemble of memories of dual events
Source: Mol Brain. 2021 Jun 29;14:102. doi: 10.1186/s13041-021-00798-3 (PMC8243579; doi:10.1186/s13041-021-00798-3)
Supplement: Supplementary file 3 — Additional file 3: Fig. S3. Seizure induced expression of IEG coupled fluorescence is described by first order consecutive kinetics: (a) Schematic of IEG induction to bicuculline administration and following its dynamics in an anesthetised transgenic mouse through in-vivo imaging of the retrosplenial cortex (RSc). (b) Select regions of interest centred around cells #39, #41, #46 and #5, are arranged as time series show the change in fluorescence across the entire cell nuclei. (c) Quantitative measure of cellular response extracted from the time series images through custom built software for four representative cells are shown as open circles. The open circles are obtained using the workflow (SFig. 1) and represent the activity of a neuron at a given time. The red line is the fit of this activity to Eq. 1. Blue dotted line extends the solid red line to the activity of the cell outside of the imaging time frame as predicted by our model. See table 3 for fit parameter details. [file 13041_2021_798_MOESM3_ESM.docx]

**
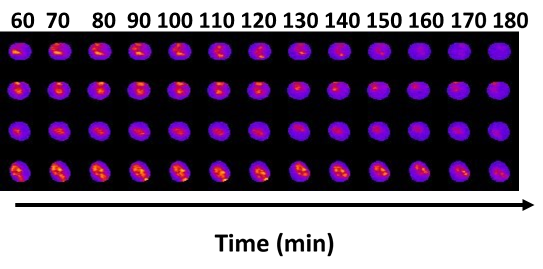
**
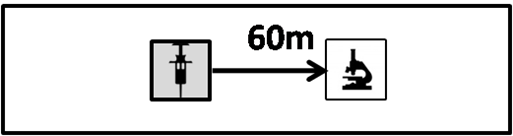
**S3**

**(b)**

**(a)**

**(c)**

**Table 3**

| **Fig No.** | **Cell No.** | **Amplitude**  **(D.U.)** | **Error in Amplitude** | **k_f_**  **(min^-1^)** | **Error in k_f_** | **K_d_**  **(min^-1^)** | **Error in k_d_** | **Adj-R-sq** | **AIC** |
| --- | --- | --- | --- | --- | --- | --- | --- | --- | --- |
| SFig S3 (c) | Cell #39 | 2640 | 39.68309 | 0.04605 | 0.00367 | 0.00176 | 1.41E-04 | 0.97354 | 85.43579 |
| SFig S3  (c) | Cell #41 | 2576 | 37.18148 | 0.04616 | 0.00355 | 0.00168 | 1.34E-04 | 0.97265 | 83.87009 |
| SFig S3 (c) | Cell #46 | 2262 | 32.49374 | 0.05358 | 0.00587 | 0.0011 | 1.30E-04 | 0.93118 | 84.81639 |
| SFig S3 (c) | Cell #05 | 2888 | 76.33026 | 0.04576 | 0.00633 | 0.00183 | 2.49E-04 | 0.92799 | 102.19985 |

**Table 3: Summary of fit parameters of cfos-egfp expression in response to seizure data fit to equation 1.**
